# Cigarette Smoking among Medical Students from the Western Balkan

**DOI:** 10.3390/ijerph19053055

**Published:** 2022-03-05

**Authors:** Miloš Ilić, Maja Grujičić, Budimka Novaković, Aleksandra Vrkatić, Zagorka Lozanov-Crvenković

**Affiliations:** 1 Department of Pharmacy, Faculty of Medicine, University of Novi Sad, Hajduk Veljkova 3, 21000 Novi Sad, Serbia; budimka.novakovic@mf.uns.ac.rs (B.N.); vrkaticaleksandra@gmail.com (A.V.); 2 Department of General Education Subjects, Faculty of Medicine, University of Novi Sad, Hajduk Veljkova 3, 21000 Novi Sad, Serbia; maja.grujicic@mf.uns.ac.rs; 3 Department of Mathematics and Informatics, Faculty of Sciences, University of Novi Sad, Trg Dositeja Obradovića 3, 21000 Novi Sad, Serbia; zlc@dmi.uns.ac.rs

**Keywords:** smoking, medical students, young adults, risk factors, public health, Western Balkans

## Abstract

University student’s smoking is a significant public health problem. It is estimated that, globally, every fifth medical student is a smoker. So far, no research dealing with cigarette smoking among medical students has been conducted in the countries of the Western Balkans. The aim of this study was to examine the prevalence and risk factors of cigarette smoking among Western Balkans medical students. A cross-sectional study was conducted among 2452 students from 14 medical faculties in the Western Balkans (Republic of Slovenia, Republic of Croatia, Bosnia and Herzegovina, Republic of North Macedonia and Republic of Serbia). The data were gathered through an online survey. There were significantly more non-smokers than smokers among medical students. Only gender and parents smoking status were statistically significantly associated with students smoking status. The smokers were more often male students, who lived in urban areas prior their studies, and whose parents were both smokers. With the aim of monitoring and enhancing student population health, it is necessary for public health activists and health officials to continually survey the students’ smoking status in order to recognize the smoking influencing factors, and form and take on appropriate activities to improve the prevention of cigarette smoking among students, as well as motivate those who smoke to give up smoking, which would contribute to improving the health of the student population.

## 1. Introduction

The World Health Organization (WHO) defines smoking as the occasional or permanent use of tobacco or tobacco products [1]. Smoking negatively affects the health and quality of life of the smoker and people around him, and is considered an addiction classified into a group of mental and behavioral disorders [1,2]. Smoking is the second leading risk factor (9%) for developing of mass non-communicable diseases (NCD) (cardiovascular diseases, chronic obstructive pulmonary disease, malignant diseases, etc.) and premature death globally [3]. Centers for Disease Control and Prevention (CDC) data show that second-hand smokers have a 30% higher risk of developing lung cancer and cardiovascular disease compared to people who are not exposed to tobacco emissions [4]. Attributable disability-adjusted life-years (DALYs) are highest for tobacco smoking (170.9 million DALYs) compared to all other factors affecting the global disease burden [5].

Prevalence of cigarette smoking in relation to gender and education level for the general population in 2019 was higher in the countries of the Western Balkans region compared to the European Union average (Table 1) [6,7,8].

According to the WHO, as of 2016, in all countries in the Western Balkans region, a ban on smoking was regulated by legislation to a greater or lesser extent [9]. The regulations are mainly aimed at banning smoking in indoor public places and advertising, promotion and sponsorship of tobacco products (Table 2) [9]. With a stronger set of tobacco control policy legislations, smoking can be reduced by 25–30% within 5 years, by 32–40% within 15 years, and by 38–48% within 40 years in the Western Balkans, and in the long-term prevent the development of NCDs and premature mortality [9].

Studies show that smoking among university students is a significant public health issue [10]. Every third university student in America is an active smoker [10], while globally, every fifth medical student is a smoker [11]. Smoking among university students is positively associated with being overweight and obese, insufficient physical activity (PA), lower socioeconomic status, urban settlement, alcohol and drug abuse, negative influence of parents and peers, media, social networks, as well as easy access to tobacco products on the market [12,13,14,15]. Smoking physicians are less likely to emphasize the health effects of smoking and to recommend stopping smoking for patients opposed to non-smoking physicians, so it is especially important to monitor smoking factors and conduct adequate targeted public health actions for medical students [11,16].

So far, no research dealing with cigarette smoking among medical students has been conducted in the countries of the Western Balkans (Republic of Slovenia, Republic of Croatia, Bosnia and Herzegovina, Republic of North Macedonia and Republic of Serbia).

The aim of this study was to examine the prevalence and risk factors of cigarette smoking among Western Balkans medical students.

## 2. Materials and Methods

### 2.1. Study Design and Population

A cross-sectional study was conducted among 2452 students from 14 medical faculties in Western Balkans: Faculty of Medicine of the University of Ljubljana (Republic of Slovenia); Faculty of Pharmacy and Biochemistry of the University of Zagreb, Faculty of Medicine of the University of Rijeka (Republic of Croatia); Faculty of Medicine of the University of Sarajevo, Faculty of Pharmacy of the University of Sarajevo, Faculty of Health Studies of the University of Sarajevo, Faculty of Medicine of the University of Zenica, Faculty of Pharmacy of the University of Mostar, Faculty of Health Studies of the University of Mostar (Bosnia and Herzegovina); Faculty of Medicine of the University “St. Cyril and Methodius” Skopje, Faculty of Pharmacy of the University “St. Cyril and Methodius” Skopje (Republic of North Macedonia); Faculty of Pharmacy of the University of Belgrade, Faculty of Medicine Novi Sad of the University of Novi Sad, Faculty of Pharmacy of the University of Business Academy Novi Sad (Republic of Serbia), in the period from November 2019 to February 2020. A convenience sampling of medical faculties was used.

### 2.2. Study Questionnaire

An online Google form survey accessible from any device was used for the data gathering. The questionnaire was uploaded and forwarded to the students via student representatives by using emails, social network profiles (Facebook), or by posting on the faculty website. The examinees were allowed to pull out of the survey on a voluntary basis at any phase before submitting it. When they finished the survey, the forms were sent to a database to be downloaded as a Microsoft Excel sheet. Only the answers from fully completed questionnaires entered the database and they were taken in the further analyses. Research method guaranteed the privacy of respondents.

### 2.3. Variables

The survey consisted of two parts. The first part of the survey contained introductory questions (which faculty students attended, their gender, year of study, body height, body weight, household income, type of settlement they lived in before starting university education), while the second part of the questionnaire contained the questions about the average daily level of PA, alcohol consumption, parents smoking status and student smoking status.

When asked about the smoking status and the number of cigarettes smoked, students answered by choosing one of the six offered answers: I am not a smoker, I smoke occasionally, up to 5 cigarettes a day, 5–10 cigarettes a day, 11–20 cigarettes a day, or more than 20 cigarettes a day. Based on the answers, students were grouped into “smokers” (I smoke occasionally, up to 5 cigarettes a day, 5–10 cigarettes a day, 11–20 cigarettes a day and more than 20 cigarettes a day) and “non-smokers” (I am not a smoker).

Medical students were grouped based on years of study into two categories, 1–3-year students and 4–6-year students.

Self-reported data on body height and weight was used to calculate body mass index (BMI). The classification was performed based on the WHO recommendation: underweight for BMI values less than 18.50 kg/m^2^, normal weight for values 18.50–24.99 kg/m^2^, overweight for values 25.0–29.99 kg/m^2^, and obesity for values greater and equal than 30.0 kg/m^2^.

Household income data were obtained by answering the survey question on average household income choosing one of the five offered answers: far below average, below average, average, above average, far above average. The responses far below average and below average were grouped and categorized as “below average income”, while above average and far above average responses were grouped and categorized as “above average income”.

When asked about the type of settlement they lived in before starting university education, the respondents answered one of seven offered answers: a village of up to 500 inhabitants, a village of 500 to 3000 inhabitants, a village of over 3000 inhabitants, a town of up to 20,000 inhabitants, a town of 20,000 to 100,000 inhabitants, a town of 100,000 inhabitants to 1 million inhabitants, a town of over 1 million inhabitants. The answers: village up to 500 inhabitants, village from 500 to 3000 inhabitants and village over 3000 inhabitants were grouped and categorized as “rural type settlements”, the answers town up to 20,000 inhabitants, town from 20,000 inhabitants up to 100,000 inhabitants, town from 100,000 inhabitants up to 1 million inhabitants and town over 1 million inhabitants were grouped and categorized as “urban type settlements”.

The students were to answer the question about average daily time spent engaging in PA, by checking one of five offered answers: I do not engage in regular PA, up to 30 min per day, up to 1 h per day, 1–2 h per day, or 3 or more hours per day. The responses of students being engaged in 1–2 h of PA per day, and 3 or more hours per day were grouped and categorized as “I engage in PA more than 1 h per day”.

Alcohol consumption was determined by choosing one of five offered answers: I do not drink alcohol, occasionally, on weekends, several times a week, or daily.

The students were asked if any of their parents was a smoker, which was determined by the four choice answers: mother, father, both, or neither.

### 2.4. Statistical Analysis

In statistical analysis, categorical variables were shown as frequencies and percentages. χ^2^ test was used to examine the association between categorical variables. As a measure of association, Cramer’s V was used.

The multivariable logistic regression analysis was used to analyze the impact of gender, year of study, BMI, daily level of PA, alcohol consumption, household income, type of settlement and parents smoking status on students smoking status.

As a dependent variable (student smoking status), in the model of multivariable logistic regression, the value “Smoker” (0 = No, 1 = Yes) was used.

The independent variables, in the multivariable logistic regression analysis, were as follows: gender (0 = female, 1 = male), year of study (0 = 1–3, 1 = 4–6), overweight and obese (0 = No, 1 = Yes), any daily level of physical activity (0 = No, 1 = Yes), alcohol consumption (0 = I do not drink alcohol, 1 = occasionally, on weekends, several times a week and daily), household income (0 = below average and average, 1 = above average), type of settlement (0 = rural, 1 = urban), and any parent smoking status (0 = No, 1 = Yes). All odds ratio (OR) values were adjusted.

The SPSS Statistics for Windows ver. 24 (IBM Corporation, Armonk, NY, USA) programme was used for statistical analysis. *p* value less than 0.05 was considered statistically significant.

### 2.5. Ethical Aspects of the Research

The study was conducted according to the guidelines of the Declaration of Helsinki. The Ethics Committees/Commissions of the faculties that participated in research gave an opinion that approval of the committees/commissions was not required, as the research did not include invasive methods and did not violate the privacy of respondents.

## 3. Results

The sample included 2015 (82.2%) female students and 437 (17.8%) male students.

There was statistically significant difference in terms of gender structure between medical faculties (χ^2^ = 50.032, *p* < 0.001, fi = 0.143) (Table 3). The highest percentage of female students was at the Faculty of Medicine of the University of Rijeka, Republic of Croatia (91.1%), while the highest percentage of male students was at the Faculty of Medicine of the University of Zenica, Bosnia and Herzegovina (26.3%), compared to other faculties.

There was a statistically significant difference in the distribution of students in terms of year of study between medical faculties (χ^2^ = 118.315, *p* < 0.001, fi = 0.22) (Table 4). The highest percentage of 1–3-year students was at the Faculty of Health Studies of the University of Mostar, Bosnia and Herzegovina (94.3%), while the highest percentage of 4–6-year students was at the Faculty of Medicine of the University of Ljubljana, Republic of Slovenia (47.7%), in comparison to other faculties. 

There was a statistically significant difference in the prevalence of smokers between medical faculties (χ^2^ = 36.35, *p* = 0.001, fi = 0.122) (Table 5). The largest percentage of student smokers was present at the Faculty of Pharmacy of the University of Belgrade, Republic of Serbia (32.3%), and the smallest was present at the Faculty of Health Studies of the University of Sarajevo, Bosnia and Herzegovina (13.9%), compared to other faculties.

There was a statistically significant difference in the percentage of female smokers between medical faculties (χ^2^ = 40.157, *p* < 0.001, fi = 0.141) (not shown in tables), with the highest percentage of female smokers at the Faculty of Pharmacy of the University of Belgrade, Republic of Serbia (31.3%), and the lowest at the Faculty of Medicine of the University of Zenica, Bosnia and Herzegovina (10.6%) (Table 6). There was no statistically significant difference in the percentage of male smokers between medical faculties (χ^2^ = 14.784, *p* = 0.321, fi = 0.184) (not shown in tables).

If each faculty was observed individually, only at the Faculty of Medicine of the University of Zenica, Bosnia and Herzegovina (χ^2^ = 7.482, *p* = 0.006, fi = 0.191), the Faculty of Pharmacy of the University of Mostar, Bosnia and Herzegovina (χ^2^ = 5.989, *p* = 0.014, fi = 0.233), the Faculty of Pharmacy of the University “St. Cyril and Methodius” Skopje, Republic of North Macedonia (χ^2^ = 7.46, *p* = 0.006, fi = 0.242) and the Faculty of Medicine Novi Sad of the University of Novi Sad, Republic of Serbia (χ^2^ = 7.164, *p* = 0.007, fi = 0.139) was there a statistically significant association between gender and student smoking status (Table 6). At the Faculty of Medicine of the University of Zenica, Bosnia and Herzegovina, a significantly higher percentage of male students were smokers compared to female students (25.9% vs. 10.6%), as well as at the Faculty of Pharmacy of the University of Mostar, Bosnia and Herzegovina (42.9% vs. 18.0%), the Faculty of Pharmacy of the University “St. Cyril and Methodius” Skopje, Republic of North Macedonia (44.4% vs. 19.0%) and the Faculty of Medicine Novi Sad of the University of Novi Sad, Republic of Serbia (32.4% vs. 18.2%).

There were more male smokers (29.5%) than female smokers (20.4%). The difference was statistically significant (χ^2^ = 17.191, *p* < 0.001, fi = 0.084) (Table 7). Students coming from urban settlements were more frequenlty smokers (24.0%), than students coming from rural settlements (17.6%) (χ^2^ = 12.55, *p* < 0.001, fi = 0.072). Students whose both parents were smokers were in significantly higher percentage (31.9%) compared to students whose at least one parent was a smoker, mother (25.1%) or father (25.6%), and students for whom neither parent was a smoker (17.8%) (χ^2^ = 37.82, *p* < 0.001, fi = 0.124). There was no statistically significant difference in the percentage of student smokers of different year of study (χ^2^ = 0.335, *p* = 0.563, fi = 0.012), BMI (χ^2^ = 7.601, *p* = 0.055, fi = 0.056), different average daily levels of PA (χ^2^ = 5.684, *p* = 0.128, fi = 0.048), alcohol consumption (χ^2^ = 2.706, *p* = 0.608, fi = 0.033) and household income (χ^2^ = 0.46, *p* = 0.790, fi = 0.014).

The model of multivariable logistic regression analysis showed that only gender and parents smoking status were statistically significant associated with student smoking status (Table 8). The odds of being a smoker for male students were 1.463 times higher than for female students (95% CI: 1.013–2.113; *p* = 0.042). The odds of being smoker for students for whom at least one parent was smoker were 1.841 times higher than students whose parents were non-smokers (95% CI: 1.347–2.516; *p* < 0.001). Year of study, BMI, daily level of PA, alchohol consumption, household income and type of settlement were not statistically significant predictors of students’ smoking status.

## 4. Discussion

The main results of our research show a high prevalence of smoking among medical students from the Western Balkans, which can present a major public health problem. In multivariable analyses, association of the gender and parents smoking with students smoking is emphasized and verified. However, in bivariate analysis, urban settlement was associated with students’ smoking. This study provides a basis for future prospective research that can be conducted on the impact of differences in tobacco control policy and university education on the incidence and prevalence of smoking in the student population.

Our study shows that there were significantly more female students at the Faculty of Medicine of the University of Rijeka, Republic of Croatia compared to other faculties, while at the Faculty of Medicine of the University of Zenica, Bosnia and Herzegovina there were more male students in relation to other faculties. Observed on the whole sample, female students made up 82.2% of the respondents. The European Commission’s report for 2020, which included member states of the European Union (EU) and candidate countries for EU membership, states that in most countries, the percentage of women in health care system is significantly higher than men [17]. In the Republic of Slovenia and in the Republic of Croatia, as well as in the Republic of Serbia and in Montenegro, there are more than three-fifths of women employed as health workers in comparison to males [17]. A report by the European Commission indicates that at the European level, 80% of medical students graduates are women [18].

The results of our research show that the largest percentage of student smokers was present at the Faculty of Pharmacy of the University of Belgrade, Republic of Serbia (32.3%), and the lowest percentage of student smokers was present at the Faculty of Health Studies of the University of Sarajevo, Bosnia and Herzegovina (13.9%), in relation to other faculties. A study by Warren et al. [19] conducted in European countries shows that over 30% of the medical students are smokers. Warren et al. [19] state that smoking physicians significantly diminish the negative effects of smoking on one’s health. They do not recommend smoking cessation frequently enough compared to non-smoking physicians [19]. Authors emphasize that additional education of medical students on the harmful effects on smoking would contribute to reducing and quitting cigarette consumption [19]. A study by Šljivo et al. [20] conducted in Bosnia indicates that approximately 60% of medical students were cigarette smokers, which could be considered as alarming information about the inadequacy of the health educational system. The same study points out that less than one-third of smokers received specific training on smoking cessation [20]. Ilić et al. [21] conducted a study in the Republic of Serbia and showed that 17.3% of pharmaceutical students are smokers. The reasons are unclear, although likely relating to legal regulations on cigarette sales as well as affordable prices; however, there is also insufficient knowledge of students about the hazards and consequences of cigarette smoking [21]. 

The obtained results of our study show that among all students of medical faculties, the Faculty of Pharmacy of the University of Belgrade, Republic of Serbia had the highest percentage of female smokers, while no significant difference was found in the percentage of male smokers relating to the faculty they attended. At the Faculty of Medicine of the University of Zenica, Bosnia and Herzegovina, the Faculty of Pharmacy of the University of Mostar, Bosnia and Herzegovina, the Faculty of Pharmacy of the University of “St. Cyril and Methodius” in Skopje, Republic of North Macedonia and the Faculty of Medicine Novi Sad of the University of Novi Sad, Republic of Serbia, male students smoked in significantly higher percentages compared to female students, while there was no significant difference in relation to gender at other faculties. Having taken into consideration all medical students, the result of our research also shows that there were more male than female smokers. The results of a study by Brożek et al. [22] conducted in Central and Eastern Europe encircling medical students indicate that there are more male than female smokers, similar to the results of our research. A study done by Brinker et al. [23] among nursery school students in Germany, and Šljivo et al. [20] conducted on medical students from Bosnia, show that there are more male smokers in comparison to female smokers. The authors point out that prevalence of female smokers is lower than that of male students due to smoking not being culturally and socially acceptable by women [23].

Our results imply that there was higher percentage of medical students who lived in urban areas prior to their studies who were smokers in comparison to the ones who lived in rural areas. The result of a study by Šljivo et al. [20] conducted among Bosnian medical students confirms a higher prevalence of cigarette smoking among students living in urban settlements prior to their studies than the ones living in rural settlements. The reason for more frequent cigarette smoking among urban settlements students in relation to the ones from the rural ones is explained by an easier availability of tobacco products on the market [20]. According to the results obtained by Basu et al. [24] in a study conducted in Bengal, the type of dwelling settlement was not associated with the students’ smoking status, which is not in line with the stated result of our research.

The percentage of medical students in our study whose both parents were smokers was significantly higher compared to the students with one parent who smoked and the ones with non-smoking parents. The results by Basu et al. [24] conducted in Bengal, a study by Abdulghani et al. [25] conducted in Saudi Arabia, a study by Tien Nam et al. [26] conducted in Vietnam, a study by Ghimire et al. [27] conducted in Nepal, and the study by Elamin et al. [28] conducted in Sudan all correspond to the results of our study, indicating the connection between the smoking status of the parents with the smoking status of their children-medical students. Among the medical students for whom both or one parent are smokers in relation to the students with non-smoking parents, it is clear that the parental model behaviour has been adopted, i.e., the students whose parents are smokers become smokers themselves more frequently [24,25,26,27,28]. A study by Deressa and Azazh [29] conducted in Ethiopia shows that the medical students whose fathers are smokers consume cigarettes in a higher percentage than the students whose fathers do not smoke. The authors highlight the important role of fathers on their children’s lives and that children adopt the behavioural patterns of their fathers’ authoritative figures [29].

A study by Steiner-Hofbauer et al. [30] conducted on medical students from Italy indicates that percentage of tobacco smokers was higher in second year medical students compared to sixth year medical students, which is inconsistent with results of our study that show no significant difference in the prevalence of smoking among students from lower and higher years of study. The same study suggests that a desire to reduce stress was the main reason for tobacco smoking and that medical faculty curriculum has a positive effect on students to quit smoking [30]. Similar is stated in the study by Lotrean et al. [31] conducted on Romanian medical students, indicating smoking to be more frequent among younger students and that smoking cessation programs targeting young adults, including education, counseling and support services, have a positive effect on quitting smoking. Conversely, Majra [32], in a study conducted in India, shows the increase in cigarette consumption among medical students in the course of their studying, The same study indicates that the increase of the prevalence of cigarette smoking is 4.2 times higher in female students compared to male students [32].

Similar to our research, Mansouri et al. [33], in a study conducted in Iran, indicate that there is no significant correlation between the BMI and the smoking status of medical students. However, according to the results by Rabanales-Sotos et al. [12] conducted in Peru, and Aldahash et al. [34] conducted in Saudi Arabia, BMI does have a correlation with smoking status of medical students. The results obtained in a study by Rabanales-Sotos et al. [12] have shown that medical students who are smokers have higher BMI values. Rabanales-Sotos et al. [12] point out that bad eating habits are usually followed by bad living habits such as cigarette smoking.

A study conducted by Mansouri et al. [33] among students at 22 universities in Iran, similar to our study, indicates that the different levels of physical activity are not associated with students’ smoking status. However, Mensouri et al. [33] emphasise that the results of the Iranian study involving only medical students show that the students who are smokers are mainly physically inactive, which is not in agreement with the results of our study, stating that there is no correlation between physical activity and medical students’ smoking status. The results of a study by Tien Nam et al. [26] conducted in Vietnam indicate that physically active students who go in for sports have more social interactions with their peers, which sometimes leads to peer pressure to start smoking and become smokers.

Contrary to the results of our study, Creamer et al. [35], in a study conducted on 24 Texas colleges (medical included), showed a significant positive association of drinking alcohol on weekends and smoking tobacco. The same study indicates that a tendency for risk behavior was highly associated with drinking and smoking, and that applied regulations have an impact on reducing smoking in students’ populations [35]. The study by Nasui et al. [36] conducted on medical students from Romania showed that medical students who drank alcohol in higher amounts were more frequent smokers. The authors emphasize that medical students starting university education are at risk for alcohol and cigarette abuse behaviors because of changes in lifestyle and reduced parental support [35,36]. A way to cope with stressful life events, social engagement or to enhance mood, could be the most common reasons for alcohol consumption among medical students [35,36].

A study by Ergin et al. [37] conducted in Turkey shows that there is no significant correlation between the average household income and the smoking status of medical students, which is in line with the results of our study. Nasser and Zhang [38], in their study conducted among medical students in Yemen, indicate that the average household income is related to the smoking status of medical students. The same study shows that the students living in households with higher average income are more likely to smoke in relation to the students living in households with lower average income [38]. Tien Nam et al. [26] in a study conducted among Vietnam medical students indicate that the students who consider their financial situation bad are more likely to smoke cigarettes in comparison to the students who consider their financial situation satisfactory. It is possible that some students use cigarettes to relieve the stress caused by financial problems or to cope with the life stress factors [26]. According to the CDC data, the US residents living below the poverty line, as well as the residents with lower income levels, smoke more than the general population [39]. It is emphasised that financial well-being contributes to the creation of positive attitudes of the population towards healthy living habits [39].

The results of our research show that, among medical students, only gender and parent smoking status had a statistically significant effect on their smoking status. The odds of being smoker were 1.463 times higher for male students compared to female students. In accordance with our results, the results of the research by Rodakowska et al. [40] conducted in Italy and Poland, and Šljivo et al. [20] conducted in Bosnia, indicate that male students are more likely to be smokers than female students. Significant cultural influences are cited as a possible reason for the difference in the frequency of smoking among male and female students, with smoking being socially unacceptable among women [40].

In our study, the odds of being smoker were 1.841 times higher for students for whom at least one parent was a smoker than students whose parents were non-smokers. The research by Afrashteh et al. [41] conducted in Iran, as well as a study by Patel et al. [42] conducted in Belgium, show that medical students are more likely to be smokers if their parents are smokers, similar to the result of our research. The authors emphasize the important role that the family plays in the initiation of tobacco consumption, i.e., that children usually easily adopt the behavioral patterns of the parents that can remain throughout their lives [41,42].

A study by Balogh et al. [43] conducted among medical students from Germany, Norway and Hungary and Šljivo et al. [20] conducted in Bosnia, point out there is a positive association of medical students smoking and older years of study, which is not in line with the results of our study where no statistically significant association between the year of study and the students smoking status was determinate.

Mansouri et al. [33] in their study conducted in Iran, as well as Rabanales-Sotos et al. [12] in a study conducted in Peru, indicate that the students with excessive body mass or suffering from obesity are more likely to smoke in relation to the students who are normal weight and the ones who are underweight, which is not in agrement with the result of our study that show that BMI was not a statistically significant predictor of the smoking status of medical students.

According to the Torchyan et al. [44] study conducted in Saudi Arabia, medical students who are highly physically active are more likely to be smokers, and a possible reason is that they, owing to sports, have more social interactions with people from their surroundings, which can lead to peer pressure to try cigarette smoking and it is not uncommon for them to become smokers. However, a study by Mansouri et al. [33], conducted in Iran, indicates that students who are not physically active on a regular basis are more likely to become smokers. The results of the aforementioned studies do not correspond with our research that shows that the level of physical activity among medical students did not have a statistically significant influence on their smoking status.

Alcohol consumption was associated with tobacco smoking in a study by Menon et al. [45] conducted in India on 58 colleges (medical included), which is not consistent with our results, that did not indicate that alcohol consumption was a statistically significant predictor of the students smoking status. The same study indicates that there was consistent evidence that drinking alcohol increased the risk of smoking tobacco [45]. A study by Nasui et al. [36], conducted in Romania on medical students, provides evidence that alcohol consumption was a positive predictor of smoking. Young people usually drank and smoked for social acceptance because of peer group pressures, to gain adult status and an image of strength.

A study by Pingak and Miller [46] conducted on nursing students in Indonesia, as well as a study by Poutiainen et al. [47] conducted among nursing students in Finland, similar to our results, indicate that average level of household income do not represent a statistically significant predictor of the students’ smoking status. However, a study by Mansouri et al. [33] conducted in Iran shows that medical students living in lower household income families have a greater risk of becoming smokers compared to the ones coming from higher household income families.

Šljivo et al. [20] in their study conducted in Bosnia indicate that it is highly likely for medical students living in urban areas to become smokers in contrast to the ones coming from rural areas, which is not in line with the results of our research that shows that there was not any statistically significant dwelling settlement influence on the smoking status of medical students.

Our study has several limitations. The study was conducted as a cross-sectional one, which is a snapshot of the actual situation; as a result, we could not perceive the changes over time, and therefore the conclusions about the cause-and-effect relations could not be made [48]. An online, anonymously filled-in self-administered survey was used as a data collector, which is a disadvantage of the research. In spite of our claim that the survey was anonymous and confidential, it is not possible to establish how honest the examinees were. Examinees have a tendency to avoid giving true answers [48], so the veracity of the answers cannot be confirmed. In the end, a limitation of the study is the use of a convenience sampling method to examine medical students, and since it was not a random selection of faculties, the results can not be generalized to all medical student populations in the Western Balkans. We were unable to provide exact numbers of students from each faculty (response rate) at the time of study conduction, but the percentage of surveyed students of all individual faculties certainly exceeds a representative 10%.

## 5. Conclusions

There are significantly more non-smokers than smokers among medical students. Only gender and parents smoking status are statistically significant associated with students smoking status. The smokers are more often male students, who lived in urban areas prior their studies, and whose both parents are smokers.

With the aim of monitoring and enhancing student population health, it is necessary for public health activists and health officials to continually survey the students’ smoking status in order to recognize the smoking influencing factors, and form and take on appropriate activities to improve the prevention of cigarette smoking among students, as well as motivate those who smoke to give up smoking, which would contribute to improving the health of the student population.

## Figures and Tables

**Table 1:** Prevalence of cigarette smoking in countries of Western Balkan region in relation to gender and education level.

Country	Gender	Education Level				Reference
		Less than Primary, Primary and Lower Secondary Education (Levels 0–2)	Upper Secondary and Post-Secondary Non-Tertiary Education (Levels 3 and 4)	Tertiary Education (Levels 5–8)	No Data in Relation to Education ***	
Republic of Slovenia *	Male	23.1	30.5	23.9	-	[6]
	Female	17.4	26.8	22.5	-	
Republic of Croatia *	Male	25.2	31.8	24.5	-	[6]
	Female	12.7	30.6	22.6	-	
Bosnia and Herzegovina	Male	-	-	-	56.3	[7]
	Female	-	-	-	31.6	
Republic of North Macedonia **	Male	-	-	-	50.6	[8]
	Female	-	-	-	35.4	
Republic of Serbia **	Male	32.5	37.1	25.4	-	[6]
	Female	24.8	34.4	27.3	-	
European Union	Male	29.6	33.7	21.4	-	[6]
	Female	17.2	23.1	16.8	-	

* EU member; ** EU candidate for membership; *** Tobacco prevalence in relation to gender and education level for Bosnia and Herzegovina and Republic of North Macedonia was not available on Eurostat.

**Table 2:** Tobacco control legislations in relation to countries in the Western Balkans.

Country	Tobacco Control Policy Legislations	
	Indoor Public Places	Advertising, Promotion and Sponsorship
Republic of Slovenia	Health care facilities and education facilities including universities are completely smoke-free. In government facilities, indoor offices, restaurants, cafés, pubs, bars, and public transport, designated smoking rooms with strict technical requirements are allowed under the current legislation.	Direct and indirect advertising ban.
Republic of Croatia	Health care facilities and education facilities including universities are completely smoke-free. In government facilities, indoor offices, restaurants, cafés, pubs, bars, and public transport, designated smoking rooms with strict technical requirements are allowed under the current legislation.	Direct and indirect advertising ban.
Bosnia and Herzegovina	No indoor public places are completely smoke-free. Designated smoking rooms with strict technical requirements are allowed in all indoor public places under the current legislation of both the Federation of Bosnia and Herzegovina and the Republika Srpska.	Direct and indirect advertising ban.
Republic of North Macedonia	Almost all enclosed public places are completely smoke-free (exclusion of indoor offices).	Direct and indirect advertising ban.
Republic of Serbia	Health care facilities, education facilities including universities, government facilities and public transport in Republic of Serbia are completely smoke-free. Indoor offices, restaurants, cafés, pubs, bars designated smoking rooms with strict technical requirements are allowed under the current legislation.	Direct and indirect advertising ban.

**Table 3:** Distribution of medical students (*n* = 2452) by gender in relation to the attended faculty.

Country	Faculty	Male		Female		*p* *
		n	%	n	%	
Republic of Slovenia	Faculty of Medicine of the University of Ljubljana	27	12.4	191	87.6	<0.001
Republic of Croatia	Faculty of Pharmacy and Biochemistry of the University of Zagreb	35	12.9	236	87.1	
	Faculty of Medicine of the University of Rijeka	13	8.9	133	91.1	
Bosnia and Herzegovina	Faculty of Medicine of the University of Sarajevo	22	17.2	106	82.8	
	Faculty of Pharmacy of the University of Sarajevo	26	21.5	95	78.5	
	Faculty of Health Studies of the University of Sarajevo	29	13.9	179	86.1	
	Faculty of Medicine of the University of Zenica	54	26.3	151	73.7	
	Faculty of Pharmacy of the University of Mostar	21	19.1	89	80.9	
	Faculty of Health Studies of the University of Mostar	27	25.5	79	74.5	
Republic of North Macedonia	Faculty of Medicine of the University “St. Cyril and Methodius” Skopje	46	25.7	133	74.3	
	Faculty of Pharmacy of the University “St. Cyril and Methodius” Skopje	27	21.3	100	78.7	
Republic of Serbia	Faculty of Pharmacy of the University of Belgrade	12	9.7	112	90.3	
	Faculty of Medicine Novi Sad of the University of Novi Sad	74	20.0	296	80.0	
	Faculty of Pharmacy of the University of Business Academy Novi Sad	24	17.3	115	82.7	

* *p* value calculated by using χ^2^ test for categorical variables. Significant at *p* < 0.05.

**Table 4:** Distribution of medical students (*n* = 2452) by year of study in relation to the attended faculty.

Country	Faculty	Year of Study				*p* *
		1–3		4–6		
		*n*	%	*n*	%	
Republic of Slovenia	Faculty of Medicine of the University of Ljubljana	114	52.3	104	47.7	<0.001
Republic of Croatia	Faculty of Pharmacy and Biochemistry of the University of Zagreb	172	63.5	99	36.5	
	Faculty of Medicine of the University of Rijeka	88	60.3	58	39.7	
Bosnia and Herzegovina	Faculty of Medicine of the University of Sarajevo	80	62.5	48	37.5	
	Faculty of Pharmacy of the University of Sarajevo	74	61.2	47	38.8	
	Faculty of Health Studies of the University of Sarajevo	169	81.3	39	18.8	
	Faculty of Medicine of the University of Zenica	108	52.7	97	47.3	
	Faculty of Pharmacy of the University of Mostar	76	69.1	89	30.9	
	Faculty of Health Studies of the University of Mostar	100	94.3	34	5.7	
Republic of North Macedonia	Faculty of Medicine of the University “St. Cyril and Methodius” Skopje	109	60.9	70	39.1	
	Faculty of Pharmacy of the University “St. Cyril and Methodius” Skopje	93	73.2	34	26.8	
Republic of Serbia	Faculty of Pharmacy of the University of Belgrade	85	68.5	39	31.5	
	Faculty of Medicine Novi Sad of the University of Novi Sad	261	70.5	109	29.5	
	Faculty of Pharmacy of the University of Business Academy Novi Sad	109	78.4	30	21.6	

* *p* value calculated by using χ^2^ test for categorical variables. Significant at *p* < 0.05.

**Table 5:** Smoking status of medical students for different medical faculties.

Country	Faculty	Smoking Status				*p* *
		Non-Smoker		Smoker		
		*n*	%	*n*	%	
Republic of Slovenia	Faculty of Medicine of the University of Ljubljana	155	71.1	63	28.9	0.001
Republic of Croatia	Faculty of Pharmacy and Biochemistry of the University of Zagreb	212	78.2	59	21.8	
	Faculty of Medicine of the University of Rijeka	106	72.6	40	27.4	
Bosnia and Herzegovina	Faculty of Medicine of the University of Sarajevo	104	81.3	24	18.8	
	Faculty of Pharmacy of the University of Sarajevo	91	75.2	30	24.8	
	Faculty of Health Studies of the University of Sarajevo	179	86.1	29	13.9	
	Faculty of Medicine of the University of Zenica	175	85.4	30	14.6	
	Faculty of Pharmacy of the University of Mostar	85	77.3	25	22.7	
	Faculty of Health Studies of the University of Mostar	87	82.1	19	17.9	
Republic of North Macedonia	Faculty of Medicine of the University “St. Cyril and Methodius” Skopje	144	80.4	35	19.6	
	Faculty of Pharmacy of the University “St. Cyril and Methodius” Skopje	96	75.6	31	24.4	
Republic of Serbia	Faculty of Pharmacy of the University of Belgrade	84	67.7	40	32.3	
	Faculty of Medicine Novi Sad of the University of Novi Sad	292	78.9	78	21.1	
	Faculty of Pharmacy of the University of Business Academy Novi Sad	101	72.7	38	27.3	

* *p* value calculated by using χ^2^ test for categorical variables. Significant at *p* < 0.05.

**Table 6:** Smoking status of medical students by gender in relation to the attended faculty.

Country	Faculty	Gender	Smoking Status				*p* *
			Non-Smoker		Smoker		
			*n*	%	*n*	%	
Republic of Slovenia	Faculty of Medicine of the University of Ljubljana	Male	20	74.1	7	25.9	0.716
		Female	135	70.7	56	29.3	
Republic of Croatia	Faculty of Pharmacy and Biochemistry of the University of Zagreb	Male	25	71.4	10	28.6	0.296
		Female	187	79.2	49	20.8	
	Faculty of Medicine of the University of Rijeka	Male	8	61.5	5	38.5	0.349
		Female	98	73.7	35	26.3	
Bosnia and Herzegovina	Faculty of Medicine of the University of Sarajevo	Male	15	68.2	7	31.8	0.084
		Female	89	84.0	17	16.0	
	Faculty of Pharmacy of the University of Sarajevo	Male	19	73.1	7	26.9	0.777
		Female	72	75.8	23	24.2	
	Faculty of Health Studies of the University of Sarajevo	Male	23	79.3	6	20.7	0.256
		Female	156	87.2	23	12.8	
	Faculty of Medicine of the University of Zenica	Male	40	74.1	14	25.9	0.006
		Female	135	89.4	16	10.6	
	Faculty of Pharmacy of the University of Mostar	Male	12	57.1	9	42.9	0.014
		Female	73	82.0	16	18.0	
	Faculty of Health Studies of the University of Mostar	Male	23	85.2	4	14.8	0.626
		Female	64	81.0	15	19.0	
Republic of North Macedonia	Faculty of Medicine of the University “St. Cyril and Methodius” Skopje	Male	37	80.4	9	19.6	0.998
		Female	107	80.5	26	19.5	
	Faculty of Pharmacy of the University “St. Cyril and Methodius” Skopje	Male	15	55.6	12	44.4	0.006
		Female	81	81.0	19	19.0	
Republic of Serbia	Faculty of Pharmacy of the University of Belgrade	Male	7	58.3	5	41.7	0.463
		Female	77	68.8	35	31.3	
	Faculty of Medicine Novi Sad of the University of Novi Sad	Male	50	67.6	24	32.4	0.007
		Female	242	81.8	54	18.2	
	Faculty of Pharmacy of the University of Business Academy Novi Sad	Male	14	58.3	10	41.7	0.083
		Female	87	75.7	28	24.3	

* *p* value calculated by using χ^2^ test for categorical variables. Significant at *p* < 0.05.

**Table 7:** Smoking status of medical students according to gender, year of study, body mass index (BMI), average daily level of physical activity, alcohol consumption, household income, type of settlement and parents smoking status.

Variables		Smoking Status				*p* *
		Non-Smoker		Smoker		
		*n*	%	*n*	%	
Gender	Male	308	70.5	129	29.5	< 0.001
	Female	1603	79.6	412	20.4	
Year of study	1–3	1271	77.6	367	22.4	0.563
	4–6	640	78.6	174	21.4	
BMI	Underweight	139	81.8	31	18.2	0.055
	Normal weight	1515	78.5	415	21.5	
	Overweight	218	73.9	77	26.1	
	Obese	39	68.4	18	31.6	
Avarage daily level of physical activity	I do not engage in regular physical activity	707	76.4	218	23.6	0.128
	Up to 30 min per day	392	81.0	92	19.0	
	Up to 1 h per day	403	79.6	103	20.4	
	More than 1 h per day	409	76.2	128	23.8	
Alcohol consumption	I do not drink alcohol	640	77.9	182	22.1	0.608
	Occasionally	869	79.0	231	21.0	
	On weekends	330	76.2	103	23.8	
	Several times a week	64	75.2	21	24.7	
	Daily	8	66.7	4	33.3	
Household income	Below average	214	79.6	55	20.4	0.790
	Average	1141	77.7	327	22.3	
	Above average	556	77.8	159	22.2	
Type of settlement	Rural	622	82.4	133	17.6	< 0.001
	Urban	1289	76.0	408	24.0	
Parents smoking status	Mother	280	74.9	94	25.1	< 0.001
	Father	285	74.4	98	25.6	
	Both	226	68.1	106	31.9	
	None	1120	82.2	243	17.8	

* *p* value calculated by using χ^2^ test for categorical variables. Significant at *p* < 0.05.

**Table 8:** Association between independent variables and smoking status of medical students’.

Variables	B	S.E.	*p*	OR	95% CI for OR
Gender (male vs. female)	0.380	0.188	0.042	1.463	1.013–2.113
Year of study (1–3 vs. 4–6)	−0.092	0.165	0.579	0.913	0.661–1.261
Overweight or obese (yes vs. no)	0.042	0.214	0.844	1.043	0.686–1.587
Any daily level of physical activity (yes vs. no)	−0.096	0.165	0.563	0.909	0.657–1.257
Alcohol consumption (occasionally, on weekends, several times a week and daily vs. I do not drink alcohol)	0.192	0.174	0.271	1.211	0.861–1.704
Household income (above average vs. below average and average)	0.182	0.184	0.321	1.200	0.837–1.720
Type of settlement (urban vs. rural)	−0.087	0.182	0.634	0.917	0.642–1.309
Any parent smoking status (yes vs. no)	0.610	0.159	<0.001	1.841	1.347–2.516
Constant	−1.756	0.271	<0.001	0.173	

Abbreviations: OR—odds ratio; CI—confidence interval; B—regression weight; S.E.—standard error.

## Data Availability

The data presented in this study are available on request from the corresponding author on reasonable request.
